# Procreative sex in infertile couples: the decay of pleasure? A letter to the editor

**DOI:** 10.1186/1477-7525-11-52

**Published:** 2013-04-11

**Authors:** Ashley Gadarowski

**Affiliations:** 1 Center for Social Work Education, Center for Human Sexuality Studies, Widener University, Chester, PA, USA

## Abstract

A response to Marci et al. ‘Procreative sex in infertile couples: the decay of pleasure?’

This is a reply to http://www.hqlo.com/content/pdf/1477-7525-11-52.pdf.

## To the Editor

Marci et al. recently examined personality features of infertile couples and whether it were possible to detect sexual disorders in couples currently undergoing infertility treatment [1]. With the use of the Adjective Check List (ACL), the Female Sexual Function Index (FSFI), and the International Index of Erectile Function (IIEF), the authors conclude no personality disturbances. I have found both strengths and areas that need improvement to ensure the current research is making the best known conclusions regarding the vast complexities of infertility. The authors have provided relevant information that has set the ground work which has inspired ideas for future research.

Among the strengths of the study were the requirements for participation in groups A and B, as well as the emphasis on gender differences. I found importance in the requirements used for group A. Participants in this group recently, within two months, discovered their infertility. Peterson et al. looked at the different coping styles of couples experiencing infertility and found that the majority of research was done with couples who had been defined as having strong and stable relationships [2]. The participants in group A are important to this study because it gave the researchers a chance to look at couples who are at the beginning stages of infertility. I found the participants in group B to be important to the study because they had already been under going fertility treatment. These participants were undergoing intrauterine insemination, which Lin et al. found to be psychologically distressing to some women. The final strength of the study was the inclusion of both partners because infertility is a shared experience [3]. The importance here is that we can compare and contrast the differences between experiences.

The three improvements needed for this research are due to methodology. Strategies for further consideration follow each gap. One challenge with this study is with the control group, couples who were not intentionally looking to get pregnant. In order to have a better baseline, future researchers should include couples who are intentionally trying to get pregnant. This brings me to the next gap for this study, that it should have been longitudinal. The subject pool can be gathered from participants intentionally trying to get pregnant with known and unknown fertilities. From there, follow up surveys would help gather information about whether these couples were successful. The final gap is with the Female Sexual Function Index (FSFI) and the International Index of Erectile Function (IIEF). Neither measure goes into detail about foreplay. Such detail would be of importance because the amount of time spent on foreplay may not be satisfying for couples [4]. With this knowledge we would be able to make better conclusions about those couples who are experiencing what the authors refer to as “sex by the clock” [1].
